# Intradialysis blood pressure changes among chronic kidney disease patients on maintenance haemodialysis in a tertiary hospital south - south Nigeria: a 2 year retrospective study

**DOI:** 10.11604/pamj.2019.33.91.17000

**Published:** 2019-06-06

**Authors:** Henry Ohem Okpa, Emmanuel Edet Effa, Sidney Kelechi Oparah, John Austin Chikezie, Elvis Mbu Bisong, Patrick Ntui Mbu, Daniel Emmanuel Otokpa

**Affiliations:** 1 Department of Internal Medicine, Renal Unit, University of Calabar Teaching Hospital, Calabar, Nigeria; 2 Department of Internal Medicine, University of Calabar, Calabar, Nigeria; 3 Department of Internal Medicine, Abia State University, Uturu, Nigeria; 4 Renal Unit, Abia State University Teaching Hospital, Aba, Nigeria; 5 Department of Family Medicine, University of Calabar, Calabar, Nigeria

**Keywords:** Chronic kidney disease, haemodialysis, intradialysis hypertension, intradialysis hypotension

## Abstract

**Introduction:**

Haemodialysis (HD) which is a form of renal replacement therapy commonly prescribed for patients with chronic kidney disease (CKD). However, it is not without deleterious haemodynamic responses which may occur either during or immediately after the termination of the procedure. These may include hypotension or hypertension.

**Methods:**

This was a retrospective study that reviewed chronic kidney disease (CKD) patients on maintenance haemodialysis at the renal unit of University of Calabar Teaching Hospital, Calabar, Nigeria. In all, 71 patients were reviewed but only 64 patients had complete data for analysis. Socio-demographic, clinical and biochemical data were obtained from the records in the dialysis unit.

**Results:**

There were more males 38 (59.4%) than females 26 (40.6%) in the study. The mean age was 51.71±15.43 years and 43.04±14.03years for males and females respectively. The prevalence of intradialysis hypertension 29 (45.3%) was higher than that of intradialysis hypotension 20 (31.3%) and the commonest cause of CKD requiring haemodialysis was diabetic nephropathy. The factors associated with intradialysis hypotension were lower post-dialysis systolic blood pressure (PDSBP), diastolic blood pressure (DBP), mean arterial pressure (MAP) and less number of antihypertensive medications; while the factors associated with intradialysis hypertension were higher post-dialysis systolic blood pressure (SBP), MAP, greater number of antihypertensive medications and longer duration of haemodialysis.

**Conclusion:**

Our study shows that there are several modifiable factors associated with blood pressure fluctuations among CKD patients on maintenance haemodialysis in the renal unit of the University of Calabar Teaching Hospital, Calabar.

## Introduction

Haemodialysis which is a form of renal replacement therapy is usually a treatment modality for patients with advanced chronic kidney disease. However, this therapy is not without deleterious haemodynamic responses which may occur either during or immediately after the termination of the procedure, and may include hypotension or hypertension [1, 2]. The relationship between blood pressure (BP) changes with haemodialysis among patients on maintenance haemodialysis is complex and incompletely understood [3]. Haemodialysis has been shown to lower blood pressure in most hypertensive end-stage renal disease (ESRD) patients but some patients show a paradoxical increase in BP during the procedure [4, 5]. Intradialytic hypertension is defined as an increase in blood pressure during or immediately after haemodialysis which results in post-dialysis hypertension and this has long been recognized to occur from haemodialysis procedure, yet it is often largely ignored [2]. It has been shown to affect between 5-15% of patients on maintenance haemodialysis and occurs more frequently in patients who are older, have lower dry weights, are prescribed more antihypertensive medications, and have lower serum creatinine levels [1, 2]. However, studies in Nigeria documented a prevalence of 15.2-30.9% which occurred in patients with higher pre-dialysis systolic and diastolic blood pressure [6, 7]. The pathophysiology of intradialytic hypertension is uncertain, it is likely multifactorial and includes subclinical volume overload, sympathetic overactivity, activation of the renin angiotensin system, endothelial cell dysfunction, and specific dialytic techniques [1, 2]. On the other hand, intradialytic hypotension (IDH) is defined as a decrease in systolic blood pressure by ≥20mm/Hg or a decrease in mean arterial pressure (MAP) by 10mm/Hg from pre-dialysis to post-dialysis which is associated with symptoms [8]. Cardiovascular complications of IDH include ischaemic (cardiac or neurological) events, vascular access thrombosis, dysrhythmias, and mesenteric venous infarction [9].

Intradialytic hypotension precludes the delivery of an adequate dose of dialysis, as hypotension episodes lead to the compartment effect and result in suboptimal Kt/V_urea_ [8]. Despite improvements in dialysis technology, intradialytic hypotension is a well-recognized HD complication, occurring in 10-70% of treatments, depending on the definition with an unchanged rate of about 25% of all HD sessions [10, 11]. The prevalence varies from 8.5-19.8% as documented in studies from Nigeria [6, 7, 12]. In addition, the incidence of IDH will continue to increase as an increasing number of elderly patients will develop CKD, and also due to the progressive increase in the number of diabetic patients with CKD. Patient subgroups most likely to have IDH include those with diabetic CKD, cadiovascular disease (CVD), poor nutritional status and hypoalbuminemia, uremic neuropathy or autonomic dysfunction, severe anemia, age ≥65, and pre-dialysis systolic blood pressure <100mm/Hg [8]. The exact pathogenesis of intradialytic hypotension is not clear, however, patients with CKD have defective reactivity of the resistance vessels as well as the capacitance vessels during the HD sessions [13, 14]. The studies on intradialytic blood pressure changes in Nigeria is sparse, despite the increasing rate in the level of haemodialysis due to the increasing number of dialysis centres. The few studies available were done among all patients undergoing haemodialysis [6, 7, 12] but our study focused mainly on advanced CKD patients on maintenance HD. We have previously reviewed our incident dialysis population and found a disproportionately higher number of older patients initiating haemodialysis. We hypothesize that there may be wider blood pressure fluctuations among our patient population given the likely increased severity and higher co-morbid conditions in older patients. This study aims at determining the pattern of blood pressure changes in advanced CKD patients during haemodialysis and the factors influencing such changes in the dialysis unit of University of Calabar Teaching Hospital (UCTH).

## Methods

This was a retrospective study that reviewed CKD patients who were on maintenance haemodialysis at the renal unit of the department of internal medicine, UCTH for a two-year period from January 2014 to December 2016. In all, 71 patients were reviewed but only 64 patients had complete data for analysis. Ethical approval was sought and obtained from the Health Research Ethical Committee (HREC) of the UCTH. Patients' medical records were reviewed and information regarding the patients demography, blood pressure measurements by automated machine attached to the dialysis bed, laboratory investigations and haemodialysis prescriptions were extracted from the dialysis records and entered into a data extraction template. Intradialytic hypertension was defined as follows: an increase in systolic BP, post-dialysis systolic blood pressure (SBP) >10mm/Hg from pre-dialysis to post-dialysis [3, 15], an increase in mean arterial blood pressure (MAP) ≥15mm/Hg during or immediately after hemodialysis [16]. Intradialytic hypotension was defined as follows: a decrease in systolic blood pressure by ≥20mm/Hg or a decrease in MAP by 10mm/Hg from pre-dialysis to post-dialysis associated with symptoms [8]. No significant blood pressure changes intradialysis means the pre to post-dialysis BP that does not meet the definition for intradialysis hypertension or intradialysis hypotension. For each patient, blood pressure (BP) changes during three consecutive dialysis sessions which were recorded, average systolic BP and diastolic BP pre-dialysis and post-dialysis were obtained from patients' records and MAP calculated using the formula MAP=DBP+ (SBP-DBP/3) [17]. The average values were then taken for all other parameters extracted from the dialysis records.

**The inclusion criteria:** they included patients on maintenance haemodialysis and patients aged between 18 and above.

**The study population:** the study population was divided into three. Patients with intradialysis hypotension, those with intradialysis hypertension and those with no significant blood pressure changes during dialysis. The last group was taken as control. Patients who had less than three sessions of haemodialysis were excluded.

**Data analysis:** the data obtained were analysed using Statistical Package for Social Sciences (SPSS) version 18. Continuous variables were expressed as means and standard deviations for normally distributed data. Categorical variables were expressed as percentages. Independent sample t-test, Chi-square test, ANOVA and other statistical tests were used as appropriate. P <0.05 was considered to be significant.

**Limitations of the study:** this study is not without some limitations related to the sample size and analysis. For example, we had a small sample size and some parameters such as serum albumin, cholesterol and parathyroid hormone levels were not assessed. Secondly, intradialysis blood pressure changes and their influence on even short term clinical outcomes were not assessed. Thirdly, given the retrospective nature of this present study and missing data, no conclusions regarding cause and effect can be made. Finally, the BP parameters were obtained from an average of three dialysis sessions; however, prior studies have used 1 week averages of routine dialysis BP recordings.

## Results

Table 1 shows the demographic and clinical characteristics of participants in the study. There were more were males 38 (59.4%). Majority of the participants were in the 41-60 years age category (48.4%), were on two antihypertensive medications (35.9%), had secondary education (46.9%) and were mainly business people (35.9%). The commonest cause of chronic kidney disease was diabetic nephropathy (26.6%), closely followed by hypertensive nephropathy (21.9%) and chronic glomerulonephritis (17.2%). Vascular access was mainly through the femoral vein (59.4%) and hypertension (45.3%) was the commonest intradialytic blood pressure fluctuation. Table 2 shows the intradialysis blood pressure changes with clinical and biochemical parameters in which duration of dialysis and the number antihypertensive medications are significantly longer and greater respectively in patients with intradialysis hypertension (p= 0.002 versus p= 0.01) as compared to those with intradialysis hypotension and no significant change. The changes in all other parameters were not statistically significant but a higher proportion of the patients who experienced intradialysis hypotension had diabetic nephropathy.

**Table 1:** Demographic and clinical characteristics of participants

Variables	Frequency N (%)	
**Sex**		
Male	38(59.4%)	
Female	26(40.6%)	
**Age**		
Male	51.71±15.43	
Female	43.04±14.03	p = 0.023
**Age Category**		
18 – 40	19(29.7%)	
41 – 60	31(48.4%)	
60 above	14(21.9%)	
**Educational Background**		
No formal education	3(4.7%)	
Primary	4(6.2)	
Secondary	30(46.9%)	
Tertiary	27(42.2%)	
**Occupation**		
Student	3(4.7%)	
Civil servant	16(25.0%)	
Public servant	9(14.1%)	
Business	23(35.9%)	
Farmer	9(14.1%)	
Retired	4(6.2%)	
**Diagnosis**		
Hypertensive Nephropathy	14(21.9%)	
Diabetic Nephropathy	17(26.6%)	
Chronic Gloerulonephritis	11(17.2%)	
Obstructive Uropathy	13(20.3%)	
Sickle Cell Nephropathy	1(1.5%)	
Toxic Nephropathy	6(9.4%)	
Polycystic Kidney Disease	2(3.1%)	
**Vascular Access**		
Femoral	38(59.4%)	
Jugular	24(37.5%)	
AV fistula	2(3.1%)	
**Intradialysis BP Changes**		
Hypotension	20(31.3%)	
Hypertension	29(45.3%)	
No Significant Change	15(23.4%)	

**Table 2:** Intradialysis blood pressure changes with clinical and biochemical parameters

Variables	Hypotension N = 20, SD±, (%)	Hypertension N = 29, SD±, (%)	No Significant Change N = 15, SD±, (%)	p- value
**Sex**				0.198
Male	15(75.0%)	16(55.2%)	7(46.7%)	
Female	5(25.0%)	13(44.8%)	8(53.3%)	
Age (years)	47.25±15.91	45.89±14.94	53.87±15.05	0.886
Weight (kg)	69.26±14.46	72.74±13.16	79.41±17.09	0.768
Sodium (mmol/l)	128.70±10.17	132.20±9.05	133.53±9.77	0.643
Creatinine (µmol/l)	836.79±347.16	840.68±493.41	731.31±345.52	0.066
PCV (%)	22.15±3.59	22.21±3.93	22.53±4.67	0.371
eGFR (mls/min)	11.22±6.43	11.93±5.59	14.29±13.62	0.238
BFR (mls/min)	275.00±75.22	303.45±66.72	283.33±67.26	0.661
UF Goal (litres)	2.70±0.88	2.76±0.96	2.23±1.13	0.267
Dialysis Duration (hrs)	3.58±0.59	3.83±0.36	3.60±0.63	0.002
No. of Sessions	8.50±13.16	9.48±8.16	6.20±9.43	0.418
PreDSPB (mmHg)	168.20±27.96	155.14±28.20	157.40±29.91	0.951
PreDDPB (mmHg)	94.20±12.62	91.10±16.88	88.67±14.37	0.410
PreDMAP (mmHg)	118.86±13.18	113.02±19.04	111.57±18.64	0.240
PostDSBP (mmHg)	135.40±32.09	181.97±28.52	153.73±28.50	0.867
PostDDBP (mmHg)	74.35±15.96	100.27±16.00	91.47±15.02	0.938
PostDMAP (mmHg)	95.03±18.99	127.51±18.17	112.23±17.46	0.839
No of Antihypertensives	2.05±0.51	4.62±0.56	2.53±0.64	0.01
**Diagnosis**				
Hypertensive Nephropathy	4(20.0%)	9(31.0%)	1(6.7%)	
Diabetic Nephropathy	5(25.0%)	6(20.7%)	6(40.0%)	0.446
Chronic GN	4(20.0%)	6(20.7%)	1(6.7%)	
Obstructive Uropathy	4(20.0%)	4(13.8%)	5(33.3%)	
Sickle cell Nephropathy	0(0.0%)	1(3.4%)	0(0.0%)	
Toxic Nephropathy	3(15.0%)	2(6.9%)	1(6.7%)	
PKD	0(0.0%)	1(3.4%)	1(6.7%)	

PCV: Packed cell volume, eGFR:Estimated glomerular filtration rate, BFR:Blood flow rate, UF:Ultrafiltration, PreDSBP:Predialysis systolic blood pressure, PreDDBP:Predialysis diastolic blood pressure, PreDMAP:Predialysis mean arterial pressure, PostDSBP:Postdialysis systolic blood pressure, PostDDBP:Postdialysis diastolic blood pressure, PostDMAP:Postdialysisi mean arterial pressure

In Table 3, the post-dialysis systolic blood pressure (DSBP), dialysis diastolic blood pressure (DDBP), and dialysis mean arterial pressure (DMAP) are significantly lower in subjects with intradialytic hypotension (p<0.05) as compared to those with intradialytic hypertension; but subjects with intradialytic hypertension had significantly greater number of antihypertensive medications. Moreso, 31.0% of the subjects with hypertensive nephropathy had intradialytic hypertension while 25.0% of the subjects with diabetic nephropathy had intradialytic hypotension but these were not statistically significant (p= 0.707). In Table 4, Post DDBP, DMAP and number of antihypertensive medications (from 2 below) are significantly lower in subjects with intradialytic hypotension (p <0.05) as compared to subjects with no significant change intradialysis. Table 5 shows the intradialysis BP fluctuations in which subjects with intradialytic hypertension had a significantly higher Post DSBP, DMAP and greater number of antihypertensive medications (p <0.05) as compared to those subjects with no significant change intradialysis.

**Table 3:** Intradialytic hypotensive and hypertensive changes with clinical and biochemical parameters

Variables	Hypotension N = 20, SD±, (%)	Hypertension N = 29, SD±, (%)	p- value
Age (years)	47.25±15.91	45.89±14.94	0.766
Weight (kg)	69.26±14.46	72.74±13.16	0.396
Sodium (mmol/l)	128.70±10.17	132.20±9.05	0.223
Creatinine (µmol/l)	836.79±347.16	840.68±493.41	0.974
PCV (%)	22.15±3.59	22.21±3.93	0.953
eGFR (mls/min)	11.22±6.43	11.93±5.59	0.690
BFR (mls/min)	275.00±75.22	303.45±66.72	0.661
UF Goal (litres)	2.70±0.88	2.76±0.96	0.826
Dialysis Duration (hrs)	3.58±0.59	3.83±0.36	0.099
No. of Sessions	8.50±13.16	9.48±8.16	0.769
PreDSPB (mmHg)	168.20±27.96	155.14±28.20	0.117
PreDDPB (mmHg)	94.20±12.62	91.10±16.88	0.466
PreDMAP (mmHg)	118.86±13.18	113.02±19.04	0.211
PostDSBP (mmHg)	135.40±32.09	181.97±28.52	0.0001
PostDDBP (mmHg)	74.35±15.96	100.27±16.00	0.0001
PostDMAP (mmHg)	95.03±18.99	127.51±18.17	0.0001
No of Antihypertensives	2.05±0.51	4.62±0.56	0.0001
**Diagnosis**			0.707
Hypertensive Nephropathy	4(20.0%)	9(31.0%)	
Diabetic Nephropathy	5(25.0%)	6(20.7%)	
Chronic GN	4(20.0%)	6(20.7%)	
Obstructive Uropathy	4(20.0%)	4(13.8%)	
Sickle cell Nephropathy	0(0.0%)	1(3.4%)	
Toxic Nephropathy	3(15.0%)	2(6.9%)	
PKD	0(0.0%)	1(3.4%)	

PCV: Packed cell volume, eGFR: Estimated glomerular filtration rate, BFR: Blood flow rate, UF: Ultrafiltration, PreDSBP: Predialysis systolic blood pressure, PreDDBP: Predialysis diastolic blood pressure, PreDMAP: Predialysis mean arterial pressure, PostDSBP: Postdialysis systolic blood pressure, PostDDBP: Postdialysis diastolic blood pressure, PostDMAP: Postdialysisi mean arterial pressure

**Table 4:** Intradialytic BP fluctuations between patients with hypotension and no significant change

Variables	Hypotension N = 20, SD±, (%)	No Significant Change N = 15, SD±, (%)	p- value
Age (years)	47.25±15.91	53.87±15.05	0.219
Weight (kg)	69.26±14.46	79.41±17.09	0.074
Sodium (mmol/l)	128.70±10.17	133.53±9.77	0.165
Creatinine (µmol/l)	836.79±347.16	731.31±345.52	0.379
PCV (%)	22.15±3.59	22.53±4.67	0.793
eGFR (mls/min)	11.22±6.43	14.29±13.62	0.429
BFR (mls/min)	275.00±75.22	283.33±67.26	0.733
UF Goal (litres)	2.70±0.88	2.23±1.13	0.197
Dialysis Duration (hrs)	3.58±0.59	3.60±0.63	0.906
No. of Sessions	8.50±13.16	6.20±9.43	0.551
PreDSPB (mmHg)	168.20±27.96	157.40±29.91	0.286
PreDDPB (mmHg)	94.20±12.62	88.67±14.37	0.245
PreDMAP (mmHg)	118.86±13.18	111.57±18.64	0.209
PostDSBP (mmHg)	135.40±32.09	153.73±28.50	0.084
PostDDBP (mmHg)	74.35±15.96	91.47±15.02	0.003
PostDMAP (mmHg)	95.03±18.99	112.23±17.46	0.009
No of Antihypertensives	2.05±0.51	2.53±0.64	0.023
**Diagnosis**			0.129
Hypertensive Nephropathy	4(20.0%)	1(7.1%)	
Diabetic Nephropathy	5(25.0%)	6(42.9%)	
Chronic GN	4(20.0%)	1(7.1%)	
Obstructive Uropathy	4(20.0%)	5(35.7%)	
Sickle cell Nephropathy	0(0.0%)	0(0.0%)	
Toxic Nephropathy	3(15.0%)	0(0.0%)	
PKD	0(0.0%)	1(7.1%)	

PCV: packed cell volume, eGFR: estimated glomerular filtration rate, BFR: blood flow rate,

UF: ultrafiltration, PreDSBP: predialysis systolic blood pressure, PreDDBP: predialysis diastolic blood pressure, PreDMAP: predialysis mean arterial pressure, Post DSBP: post-dialysis systolic blood pressure, PostDDBP: post-dialysis diastolic blood pressure, PostDMAP: post-dialysisi mean arterial pressure

**Table 5:** Intradialytic BP fluctuations between patients with hypertension and no significant change

Value	Hypertension N = 29, SD±, (%)	No Significant Change N = 15, SD±, (%)	p- value
Age (years)	45.89±14.94	53.87±15.05	0.106
Weight (kg)	72.74±13.16	79.41±17.09	0.199
Sodium (mmol/l)	132.20±9.05	133.53±9.77	0.664
Creatinine (µmol/l)	840.68±493.41	731.31±345.52	0.398
PCV (%)	22.21±3.93	22.53±4.67	0.823
eGFR (mls/min)	11.93±5.59	14.29±13.62	0.529
BFR (mls/min)	303.45±66.72	283.33±67.26	0.354
UF Goal (litres)	2.76±0.96	2.23±1.13	0.138
Dialysis Duration (hrs)	3.83±0.36	3.60±0.63	0.213
No. of Sessions	9.48±8.16	6.20±9.43	0.263
PreDSPB (mmHg)	155.14±28.20	157.40±29.91	0.810
PreDDPB (mmHg)	91.10±16.88	88.67±14.37	0.619
PreDMAP (mmHg)	113.02±19.04	111.57±18.64	0.810
PostDSBP (mmHg)	181.97±28.52	153.73±28.50	0.004
PostDDBP (mmHg)	100.27±16.00	91.47±15.02	0.081
PostDMAP (mmHg)	127.51±18.17	112.23±17.46	0.011
No of Antihypertensives	4.62±0.56	2.53±0.64	0.001
**Diagnosis**			0.196
Hypertensive Nephropathy	9(31.0%)	1(6.7%)	
Diabetic Nephropathy	6(20.7%)	6(40.0%)	
Chronic GN	6(20.7%)	1(6.7%)	
Obstructive Uropathy	4(13.8%)	5(33.3%)	
Sickle cell Nephropathy	1(3.4%)	0(0.0%)	
Toxic Nephropathy	2(6.9%)	1(6.7%)	
PKD	1(3.4%)	1(6.7%)	

PCV: packed cell volume, eGFR: estimated glomerular filtration rate, BFR: blood flow rate, UF: ultrafiltration, PreDSBP: pre-dialysis systolic blood pressure, PreDDBP: pre-dialysis diastolic blood pressure, PreDMAP: pre-dialysis mean arterial pressure, PostDSBP: post-dialysis systolic blood pressure, PostDDBP: post-dialysis diastolic blood pressure, PostDMAP: post-dialysisi mean arterial pressure

## Discussion

This study has shown that the duration of dialysis as well as an increased number of antihypertensive medication use significantly affect blood pressure changes in an advanced CKD cohort with a higher frequency of diabetic patients. There were more males with higher mean age, which portrays the tendency for the development of CKD in older males and the commonest cause of CKD in patients on maintenance HD is diabetic nephropathy. These findings are in keeping with other studies [18-21]. Furthermore, intradialysis hypertension has appeared more frequent than intradialysis hypotension. This finding is at variance with other studies where the prevalence of intradialysis hypotension was higher [1-3] but this is in conformity with results from our environment [6, 7]. The difference in this finding may be due to the small sample size we had as compared to the larger sample sizes in the other studies earlier mentioned [1-3]. It may also be related to more advanced CKD and poorly functioning hearts as well as autonomic neuropathy related to uraemia in our subjects. In addition, subjects with intradialysis hypotension were younger and had higher levels of serum creatinine when compared with their counterparts with no significant change intradialysis. This is similar to findings in other studies although in our study these parameters were not statistically significant. [1-3].

Our study demonstrates that haemodynamic responses during haemodialysis were associated with pre-dialysis or post-dialysis systolic BP, diastolic BP or MAP. These changes have been demonstrated in other studies to be strongly associated with hospitalization or death. [2, 3]. However, our study may suggest that haemodynamic responses to haemodialysis may be used in identifying patients at increased risk of important short-term clinical events due to the intradialysis blood pressure changes observed. Stidley *et al.* in their investigation of incident hemodialysis patients, showed that elevated pre-dialysis SBP (>160mm/Hg) was associated with lower mortality, and low post-dialysis SBP (<110mm/Hg) was associated with increased mortality [22] but a study by Foley *et al.* found that neither pre-dialysis nor post-dialysis SBP was significantly associated with all-cause mortality after controlling for demographics, comorbid conditions, and percentage of interdialytic weight gain [23]. In this present study, subjects with intradialysis hypotension had higher pre-dialysis systolic BP, diastolic BP and MAP, with lower post-dialysis systolic, diastolic BP and MAP, and were on less antihypertensive medications when compared with those with increased blood pressure and no significant change intradialysis. However, these parameters were not statistically significant except for the less number of antihypertensive medications. In addition, when subjects with intradialyis hypotension is compared to those with intradialysis hypertension alone or with those with unchanged intradialysis blood pressure alone; those with hypotension had lower post-dialysis systolic BP, diastolic BP and MAP, and these parameters were statistically significant.

Our study showed that the duration of HD is longer in subjects with intradialysis hypertension, although hypotension has been shown to be associated with longer duration of HD [24]. Our finding probably reflects current understanding of the pathophysiologic mechanism which links failure to lower BP with dialysis, enhanced renin-angiotensin system and/or increased sympathetic nervous system activity in response to decreases in blood volume [8]. Furthermore, intradialytic hypertension may be associated with lower dry weights of the patients but our study showed a higher dry weight. Our patients pay out of pocket for dialysis and are consequently generally under dialysed. Interestingly a number of studies supported our findings but for a different reason. Patients who do not reach target dry weights are thought to be less likely to respond to HD with an appropriate lowering of BP [25, 26]. Also, the subjects with intradialysis hypertension had higher post-dialysis SBP, MAP and greater number of antihypertensive medications when compared with subjects that had no significant blood pressure changes during dialysis. This is in keeping with findings from other studies, but the study by Buren *et al.* further evaluated the relationship between intradialytic hypertension and interdialytic ambulatory blood pressure [3, 27].

## Conclusion

In conclusion, intradialysis hypotension and intradialysis hypertension are present in 31.3% and 45.3% of patients on maintenance haemodialysis respectively. On one hand, the factors associated with intradialysis hypotension were lower post-dialysis SBP, DBP, MAP and less number of antihypertensive medications, while on the other hand the factors associated with intradialysis hypertension were higher post-dialysis SBP, MAP, greater number of antihypertensive medications and longer duration of heamodialysis.

### What is known about this topic

- Intradialysis blood pressure fluctuations such as hypotension and hypertension are common complications during haemodialysis;
- Risk factors for intradialysis hypotension includes diabetic CKD, CVD, severe anemia, age ≥65, and predialysis systolic blood pressure <100mm/Hg;
- On the other hand, the risk factors for intradialysis hypertension include older patients, lower dry weights, prescription of more antihypertensive medications, and lower serum creatinine levels.

### What this study adds

- This study investigates intradialysis blood pressure fluctuations among advanced CKD patients on maintenance HD;
- Longer duration of haemodialysis is a risk factor for intradialysis hypertension.

## Competing interests

The authors declare no competing interests.
